# Ramadan Observance Exacerbated the Negative Effects of COVID-19 Lockdown on Sleep and Training Behaviors: A International Survey on 1,681 Muslim Athletes

**DOI:** 10.3389/fnut.2022.925092

**Published:** 2022-06-30

**Authors:** Mohamed Romdhani, Achraf Ammar, Khaled Trabelsi, Hamdi Chtourou, Jacopo A. Vitale, Liwa Masmoudi, Mathieu Nédélec, Dale E. Rae, Ramzi A. Al Horani, Helmi Ben Saad, Nicola Bragazzi, Gürhan Dönmez, Ismail Dergaa, Tarak Driss, Abdulaziz Farooq, Omar Hammouda, Nesrine Harroum, Bahar Hassanmirzaei, Karim Khalladi, Syrine Khemila, Leonardo Jose Mataruna-Dos-Santos, Imen Moussa-Chamari, Iñigo Mujika, Hussein Muñoz Helú, Amin Norouzi Fashkhami, Laisa Liane Paineiras-Domingos, Mehrshad Rahbari Khaneghah, Yoshitomo Saita, Maher Souabni, Nizar Souissi, Jad Adrian Washif, Johanna Weber, Piotr Zmijewski, Lee Taylor, Sergio Garbarino, Karim Chamari

**Affiliations:** ^1^High Institute of Sport and Physical Education, University of Sfax, Sfax, Tunisia; ^2^Physical Activity, Sport and Health, UR18JS01, National Observatory of Sports, Tunis, Tunisia; ^3^Institute of Sport Science, Otto von Guericke University, Magdeburg, Germany; ^4^Department of Training and Movement Science, Institute of Sport Science, Johannes Gutenberg University Mainz, Mainz, Germany; ^5^Research Laboratory: Education, Motricity, Sport and Health, EM2S, LR19JS01, University of Sfax, Sfax, Tunisia; ^6^IRCCS Istituto Ortopedico Galeazzi, Milan, Italy; ^7^The French National Institute of Sport (INSEP), Research Unit, Laboratory Sport, Expertise and Performance (EA7370), Paris, France; ^8^Division of Exercise Science and Sports Medicine, Department of Human Biology, Faculty of Health Sciences, University of Cape Town, Cape Town, South Africa; ^9^Department of Exercise Science, Yarmouk University, Irbid, Jordan; ^10^Laboratoire de Recherche (LR12SP09) “Insuffisance Cardiaque” Sousse, Faculté de Médecine de Sousse, Hôpital Farhat Hached, Université de Sousse, Sousse, Tunisia; ^11^Department of Health Sciences, Postgraduate School of Public Health, University of Genoa, Genoa, Italy; ^12^Laboratory for Industrial and Applied Mathematics, Department of Mathematics and Statistics, York University, Toronto, ON, Canada; ^13^Department of Sports Medicine, Hacettepe University School of Medicine, Ankara, Turkey; ^14^PHCC, Primary Health Care Corporation, Doha, Qatar; ^15^Interdisciplinary Laboratory in Neurosciences, Physiology and Psychology: Physical Activity, Health and Learning (LINP2), UFR STAPS (Faculty of Sport Sciences), UPL, Paris Nanterre University, Nanterre, France; ^16^Aspetar, Orthopaedic and Sports Medicine Hospital, FIFA Medical Centre of Excellence, Doha, Qatar; ^17^Research Laboratory, Molecular Bases of Human Pathology, LR19ES13, Faculty of Medicine, University of Sfax, Sfax, Tunisia; ^18^Faculty of Medicine, School of Kinesiology and Physical Activity Science, University of Montreal, Montreal, QC, Canada; ^19^Sports Medicine Research Center, Neuroscience Institute, Tehran University of Medical Sciences, Tehran, Iran; ^20^Iran Football Medical Assessment and Rehabilitation Center, IFMARC, FIFA Medical Center of Excellence, Tehran, Iran; ^21^High Institute of Sports and Physical Education, Ksar Said, Manouba University, Manouba, Tunisia; ^22^Department of Sport Management, Faculty of Management, Canadian University Dubai, Dubai, United Arab Emirates; ^23^Coventry University – Centre for Trust, Peace and Social Relation, Coventry, United Kingdom; ^24^Physical Education Department, College of Education, Qatar University, Doha, Qatar; ^25^Department of Physiology, Faculty of Medicine and Nursing, University of the Basque Country, Leioa, Spain; ^26^Exercise Science Laboratory, Faculty of Medicine, School of Kinesiology Universidad Finis Terrae, Santiago, Chile; ^27^Department of Economic-Administrative Sciences, Universidad Autónoma de Occidente, Culiacán, Mexico; ^28^Esteghlal Physiotherapy Clinic, EPC, Tehran, Iran; ^29^Programa de Pós-graduação em Ciências Médicas, Universidade do Estado do Rio de Janeiro, Rio de Janeiro, Brazil; ^30^Departamento de Fisioterapia, Instituto Multidisciplinar de Reabilitação e Saúde, Universidade Federal da Bahia, Salvador, Brazil; ^31^Department of Sports and Regenerative Medicine, Juntendo University, Tokyo, Japan; ^32^Sports Performance Division, National Sports Institute of Malaysia, Kuala Lumpur, Malaysia; ^33^Neurocognition and Action – Biomechanics, Bielefeld University, Bielefeld, Germany; ^34^Institute for Sports Science, Christian-Albrechts-University of Kiel, Kiel, Germany; ^35^Józef Piłsudski University of Physical Education in Warsaw, Warsaw, Poland; ^36^National Center for Sport and Exercise Medicine (NCSEM), School of Sport, Exercise and Health Sciences, Loughborough University, Loughborough, United Kingdom; ^37^Sport and Exercise Discipline Group, Faculty of Health, University of Technology Sydney (UTS), Sydney, NSW, Australia; ^38^Department of Neuroscience, Rehabilitation, Ophthalmology, Genetics and Maternal-Infantile Sciences, University of Genoa, Genoa, Italy; ^39^Post-graduate School of Occupational Medicine, Università Cattolica del Sacro Cuore, Rome, Italy

**Keywords:** confinement, pandemic, religious fasting, sleep-wake pattern, training load

## Abstract

**Objective:**

Disrupted sleep and training behaviors in athletes have been reported during the COVID-19 pandemic. We aimed at investigating the combined effects of Ramadan observance and COVID-19 related lockdown in Muslim athletes.

**Methods:**

From an international sample of athletes (*n* = 3,911), 1,681 Muslim athletes (from 44 countries; 25.1 ± 8.7 years, 38% females, 41% elite, 51% team sport athletes) answered a retrospective, cross-sectional questionnaire relating to their behavioral habits pre- and during- COVID-19 lockdown, including: (**i**) Pittsburgh sleep quality index (PSQI); (**ii**) insomnia severity index (ISI); (**iii**) bespoke questions about training, napping, and eating behaviors, and (**iv**) questions related to training and sleep behaviors during-lockdown and Ramadan compared to lockdown outside of Ramadan. The survey was disseminated predominately through social media, opening 8 July and closing 30 September 2020.

**Results:**

The lockdown reduced sleep quality and increased insomnia severity (both *p* < 0.001). Compared to non-Muslim (*n* = 2,230), Muslim athletes reported higher PSQI and ISI scores during-lockdown (both *p* < 0.001), but not pre-lockdown (*p* > 0.05). Muslim athletes reported longer (*p* < 0.001; *d* = 0.29) and later (*p* < 0.001; *d* = 0.14) daytime naps, and an increase in late-night meals (*p* < 0.001; *d* = 0.49) during- compared to pre-lockdown, associated with lower sleep quality (all p < 0.001). Both sleep quality (χ^2^ = 222.6; *p* < 0.001) and training volume (χ^2^ = 342.4; *p* < 0.001) were lower during-lockdown and Ramadan compared to lockdown outside of Ramadan in the Muslims athletes.

**Conclusion:**

Muslim athletes reported lower sleep quality and higher insomnia severity during- compared to pre-lockdown, and this was exacerbated by Ramadan observance. Therefore, further attention to Muslim athletes is warranted when a circadian disrupter (e.g., lockdown) occurs during Ramadan.

## Introduction

The COVID-19 induced lockdown was a major challenge to athletes’ circadian rhythms including their sleep, eating and training behaviors (1–4). Training loads were lower during-lockdown, with concomitant changes in training modalities, due to social distancing, curfew, stay-at-home or quarantine regulations (5). Some athletes displayed a lower motivation to train (1), longer and later daytime naps (4), and increased their screen use during-lockdown (2). Indeed, athletes reported fewer training sessions per week and later preferred time of day (TOD) to train (4). Furthermore, athletes reported later bed and wake times, which increased late-night eating behavior (4). These lockdown induced behavioral alterations resulted in lower sleep quality and increased insomnia severity in athletes, which were principally associated with a longer sleep onset latency (SOL) and shift toward “eveningness” (4).

During Ramadan, the ninth month of the Muslim lunar (*hijri*) calendar, pubertal healthy Muslims voluntarily abstain from eating and drinking (among others) from dawn to dusk. With the reduced energy intake during the day, continuing to train and/or compete during Ramadan can be challenging (6, 7). As meal and physical activity timing are potent *zeitgebers* (from German synchronizers), Ramadan is a potent disrupter for Muslims’ circadian rhythms in general and in particular Muslim athletes (8–10). Ramadan observance is accompanied by physiological (e.g., hypohydration, decreased body mass, and/or fat percent) (7) and behavioral (e.g., late meals, intentional nighttime wake ups, and reduced physical activities levels) (11, 12) changes that affect Muslim athletes’ training and sleeping behaviors. Indeed, recent systematic reviews reported that Ramadan observance could affect a wide range of physical performances even if many studies do not report any effect of Ramadan observance on certain physical activity performance measures (13–15). Further, in athletes, sleep quality and total sleep duration were lower (11, 16), daytime sleepiness was higher and daytime napping was longer (9, 12) during compared to before Ramadan. One particularity about the 2020 Ramadan (23 April to 23 May), is that it coincided with the early 2020 COVID-19 lockdown.

Therefore, this study aimed at investigating the effects of: (***i***) lockdown-induced behaviors on sleep quality and insomnia severity in Muslim compared to non-Muslim athletes; and (***ii***) Ramadan observance during-lockdown on training and sleep behaviors in Muslim athletes.

## Methods

### Participants and Procedures

The present study is part of an international study investigating the effects of COVID-19 lockdown on sleep quality and insomnia severity in an international sample of 3,911 athletes. The results of this project has been partly published elsewhere (4), providing full and open-access descriptions of methods and data. Muslim athletes who fasted during Ramadan 2020 (23 April to 23 May), completed a specific and additional section of this original survey. In the present study, comparisons were made between Muslim (*n* = 1,681) and non-Muslim (*n* = 2,230) athletes for the Pittsburgh sleep quality index [PSQI (17)] and insomnia severity index [ISI (18)] sections. Eligibility criteria for participants were: (***i***) ≥ 18 years of age; (***ii***) Muslim athlete (individual or team sport) of either sex; and (***iii***) participant had been under conditions of lockdown for at least 2 weeks. Informed consent was provided by participants under ethical approval from an institutional review board (Farhat HACHED Hospital, Sousse, Tunisia; IRB# FH020720), in the spirit of the Helsinki Declaration. Data were processed anonymously and according to the guidelines of the ‘‘General Data Protection Regulation.’’^1^

### Questionnaire

The full survey is provided within Supplementary Material and briefly described in Table 1, prefaced by further context and description of each the survey’s sections. This retrospective, cross-sectional and web-based questionnaire surveyed/included: (***i***) PSQI; (***ii***) ISI; (***iii***) bespoke questions about napping, training and eating behaviors in athletes who had experienced a period of lockdown, with a specific comparisons made between “during-lockdown” and the “month preceding lockdown” (i.e., baseline). Lastly, (***iv***) a Supplementary Section (to be answered only by Muslim athletes observing Ramadan) investigated training volume and sleep quality of Muslim athletes during Ramadan and lockdown compared to lockdown outside of Ramadan. The original English survey was translated into nine languages (Arabic, French, Italian, Japanese, Malay, Persian, Portuguese, Spanish and Turkish). The survey questions underwent translation and back-translation, performed by the research team (including at least one native speaker and a topic expert). Thereafter, 1% of the participants (42 athletes) responded to these newly developed questions twice (1 week apart), assessing responses’ reliability [ranging from good (*r* = 0.83) to very good (*r* = 0.97)]. The survey was launched online through social media (e.g., Facebook^®^, Twitter^®^, WhatsApp^®^, and e-mail), opening 8 July and closing 30 September 2020.

**TABLE 1:** Description of the different sections of the survey.*

Section	Description	Items
Section 1	Explanation of the study	Invited volunteers to confirm eligibility Provide consent to participate Encouraged respondents to respond as accurately as possible
Section 2	Demographic information	Sex Age Country of residence, Level of competition Sport discipline
Section 3	Training questions	Preferred TOD to train Number of training sessions per week
Section 4	Pittsburgh sleep quality index (PSQI) Insomnia severity index (ISI)	Bedtime Wake time Mid-sleep time Total sleep time Time in bed Sleep onset latency Sleep efficiency
Section 5	Napping questions	Nap timing Nap duration Nap frequency
Section 6	Nutrition- and health-related questions	Body mass Number of daily meals Late night meals Caffeine beverages
Section 7	Ramadan questions	Sleep quality and training volume during Ramadan and lockdown compared to lockdown outside of Ramadan

*The full survey questions are provided as Supplementary Material.

### Statistical Analysis

The statistical analysis was performed using SPSS (IBM Corp. IBM SPSS Statistics for Windows, Version 26. Armonk, NY: IBM Corp.) and figures were created using GraphPad Prism 8 (GraphPad Software, San Diego, CA, United States). The Shapiro Wilks test was used to assess data normality. Comparing Muslim athletes’ data from pre- to during-lockdown was assessed using the Wilcoxon Signed rank test. The magnitude of difference is reported as Cohen’s effect size (*d*) subsequently calculated, qualitatively interpreted as small (*d* < 0.5), moderate (0.5 ≤ *d* < 0.8) and large (*d* ≥ 0.8) (19). A mixed model analysis of variance (ANOVA) with repeated measure, followed by Bonferroni *post-hoc* test were used to compare Muslim to non-Muslim PSQI and ISI scores pre- and during-lockdown. Further, a single classification chi-square test was used to explore the effect of Ramadan observance on Muslim athletes’ sleep quality and training volume.

Multiple linear regression analyses were performed, based on delta variation (Δ%; % change in each variable from pre- to during-lockdown), to assess the relationships between dependent (PSQI and ISI scores) and independent (sleep, training, health and nutrition-related) variables. After checking for the assumptions (i.e., correlation between the dependent and independent variables, multicolinearity, and outliers), all the independent variables were entered into the model using a stepwise method. The R square and the semi-partial correlation coefficient squared (sr^2^: in percentage) are reported. The multiple regression model was followed by a statistical mediation based on Hayes’ guidelines (20). The magnitude of the mediation effect is expressed as a percentage according to the formula (indirect effect/total effect × 100) (20). The missing data was deleted from the pairwise comparison, and listwise deleted from the multiple regression models. All values within the text and tables are reported as mean ± standard deviation (SD). Alpha was set at *p* < 0.05.

## Results

### Participants

Of the overall sample (*n* = 3,911), 1,681 athletes answered the questions concerning Muslim athletes fasting during Ramadan. Demographic data of the overall sample, Muslim and non-Muslim athletes are presented in Table 2.

**TABLE 2:** Demographic characteristics of the overall sample, Muslim and non-Muslim athletes.

		Overall sample (*n* = 3,911)	Muslim athletes (*n* = 1,681; 43%)	Non-Muslim athletes (*n* = 2,230; 57%)
Sex	Male	54%	61%	48%
	Female	45%	38%	51%
	Not declared	1%	1%	1%
Age	Mean	25.1	25.1	25.1
	Range	18–61	18–52	18–61
	≤25	67%	58%	74%
	>25	33%	42%	26%
Sport discipline	N° disciplines	56	44	56
	Team	63%	51%	72%
	Individual	37%	49%	28%
Level of competition	Elite	37%	41%	34%
	Non-elite	63%	59%	66%
Geographic location	N° countries	49	44	46
	Asia	57%	59%	55%
	Europe	14%	16%	12%
	America	18%	1%	31%
	Africa	11%	24%	2%
	Australia	0.1%	0.1%	0.1%

### The Effects of Lockdown

**FIGURE 1:**
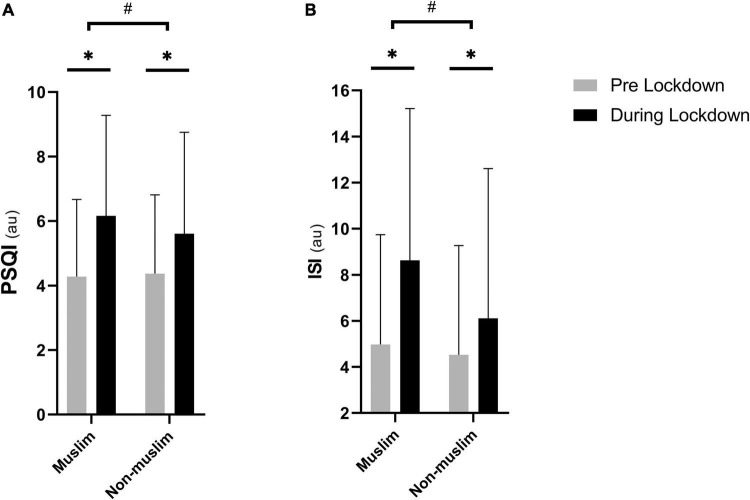
Difference between Muslim (*n* = 1,681) and non-Muslim (*n* = 2,230) athletes during- compared to pre-lockdown in **(A)** PSQI (the Pittsburgh Sleep Quality Index) and **(B)** ISI (Insomnia Severity Index). * means significant within group effect of the lockdown at *p* < 0.001. ^#^ means difference between groups during-lockdown at *p* < 0.001. Significance is assessed by mixed model ANOVA and the Bonferroni *post-hoc* test.

The lockdown reduced sleep quality and increased insomnia severity (both *p* < 0.001) in the sample of Muslim athletes. Compared to non-Muslim, Muslim athletes reported higher PSQI (Muslim: 6.2 ± 3.1; non-Muslim: 5.6 ± 3.1) and ISI (Muslim: 8.6 ± 6.6; non-Muslim: 6.1 ± 6.5) scores during-lockdown (both *p* < 0.001), while there was no difference pre-lockdown (*p* > 0.05; Figure 1). Pre-lockdown, 41% of Muslim athletes reported poor sleep quality and 10% reported very poor sleep quality; this increased during-lockdown to 68 and 32%, respectively. The proportion of athletes reporting moderate (5%) and severe (1%) insomnia pre-lockdown increased during-lockdown to 15 and 5%, respectively. The likelihood to consume food after midnight increased from 48% pre-lockdown to 71% during-lockdown (*p* < 0.001). The effect of lockdown on the selected parameters (i.e., sleep, napping, training, and eating behaviors) is presented in Table 3, with comparison made between pre- and during-lockdown.

**TABLE 3:** Changes in sleep, training, and eating behaviors from pre- to during-lockdown in Muslim athletes.

Variable	Lockdown		Z	Δ % (SD)	*p* <	*d*	MD	95% CI
	
	Pre	During						
PSQI score (a.u.)	4.3 ± 2.4	6.2 ± 3.1	22.1	83.4 (151.3)	0.001	0.68	1.9	1.7–2.1
ISI score (a.u.)	5.5 ± 4.7	8.6 ± 6.6	20.9	149 (338.4)	0.001	0.54	3.1	2.9–3.4
Preferred training TOD (hh: mm)	14:39 ± 4:37	15:25 ± 4:33	7.6	11.2 (40.7)	0.001	0.19	0:45	0:31–0:59
Training sessions (N°⋅week^–1^)	4.9 ± 2.4	3.1 ± 2.4	22.9	–29.5 (50.1)	0.001	0.75	–1.8	–1.9 to –1.7
Bedtime (hh: mm)	23:12 ± 1:58	01:08 ± 1:12	30.4	7.7 (7.5)	0.001	1.16	1:56	1:45–2:07
Wake time (hh: mm)	7:45 ± 1:47	10:55 ± 2:35	31.9	47.7 (41.9)	0.001	1.48	3:10	3:04–1:16
Mid-sleep time (hh: mm)	3:30 ± 1:15	5:59 ± 2:03	33	88.4 (96.7)	0.001	1.51	2:29	2:19–2:38
Total sleep time (min)	458 ± 71	515 ± 90	22.1	14.2 (21.9)	0.001	0.71	57	53–62
Time in bed (min)	517 ± 100	617 ± 120	27.6	21.5 (25.7)	0.001	0.91	100	93–105
Sleep efficiency (%)	90.1 ± 11.9	85.7 ± 13.8	16.9	–5.5 (8.5)	0.001	0.34	–4.4	–6 to –2.7
Sleep onset latency (min)	20.6 ± 16.4	36.5 ± 30.1	24.7	102 (131.2)	0.001	0.65	15.9	14.6–17.2
Nap frequency (N°⋅week^–1^)	1.7 ± 1.6	2.1 ± 1.8	6.9	21.1 (9.5)	0.001	0.24	0.36	0.26–0.46
Nap duration (min)	14.1 ± 20.9	21.2 ± 26.2	9.8	50.3 (25.4)	0.001	0.29	7.1	5.7–8.5
Nap timing (hh: mm)	14:26 ± 1:41	14:44 ± 1:43	5.4	11.4 (37.5)	0.001	0.14	0:18	0:13–0:23
Eat after midnight (a.u.)	0.6 ± 1.1	1.2 ± 1.3	19.3	108 (213)	0.001	0.49	0.61	0.46–0.76
Body mass (kg)	70.4 ± 14.4	72 ± 15.4	14.5	2.2 (5.7)	0.001	0.11	1.56	1.36–1.75
Meals (N°⋅day^–1^)	2.9 ± 1.2	3.2 ± 1.5	6.7	24.3 (78.1)	0.001	0.22	0.23	0.15–0.31
Caffeinated beverages (N°⋅day^–1^)	1.7 ± 1.3	1.9 ± 1.5	8.9	37.4 (100.1)	0.001	0.14	0.27	0.21–0.34

*Wilcoxon signed rank test was used to compare variables measured pre- and during-lockdown. a.u., arbitrary unit; d, Cohen’s effect size; h, hour; ISI, Insomnia Severity Index; kg, Kilogram; MD, mean difference; min, minutes; N°, number; p, probability; PSQI, Pittsburgh Sleep Quality Index; SD, standard deviation; TOD, time-of-day; 95% CI, 95% Confidence interval; **Δ** %, delta variation calculated according to the formula [100 × (during-lockdown – pre-lockdown)/pre-lockdown].*

### Multiple Regression

**FIGURE 2:**
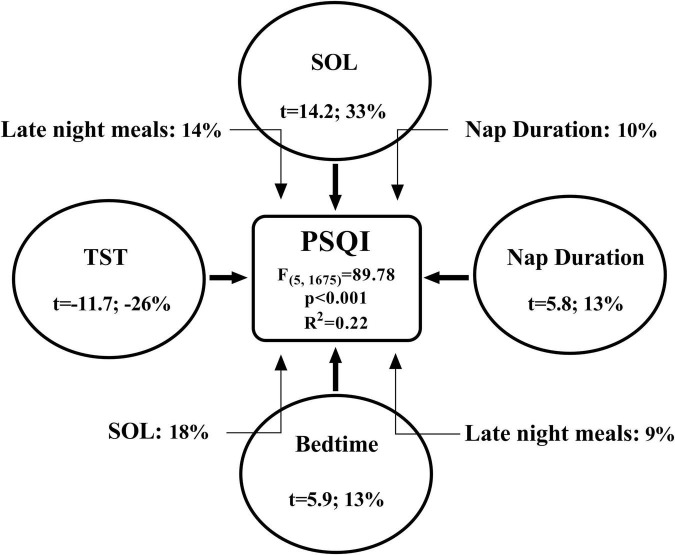
The bold arrows present the direct effect (multiple regression) and the light arrows present the indirect effects (mediation) of different independent variables on the dependent variable. The increase in PSQI (Pittsburgh Sleep Quality Index) score from pre- to during-lockdown (i.e., Δ%) was directly associated to the longer SOL (sleep onset latency) and daytime nap duration, later bedtime and reversely associated to TST (total sleep time). The magnitude of the association between independent and dependent variable is expressed as the semi-partial correlation coefficient squared (i.e., in percentages; the unique contribution of each independent variable within the model). The association between later bedtime and higher PSQI scores during-lockdown was mediated by the increased late night meals and longer SOL. Also, the increased late night meals and longer daytime nap duration mediated the relationship between longer SOL and higher PSQI scores. The magnitude of the mediation effect is expressed as percentages according to the formula (indirect effect/total effect × 100).

**FIGURE 3:**
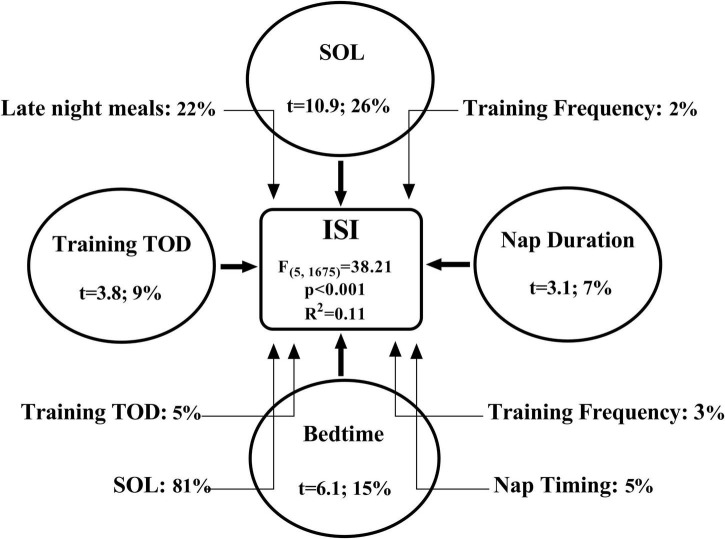
The bold arrows present the direct effect (multiple regression) and the light arrows present the indirect effects (mediation) of different independent variables on the dependent variable. The increase in ISI (Insomnia Severity Index) score from pre- to during-lockdown (i.e., Δ%) was directly associated to the longer SOL (sleep onset latency) and daytime nap duration, and later bedtime and preferred time of day to train. The magnitude of the association between independent and dependent variable is expressed as the semi-partial correlation coefficient squared (i.e., in percentages; the unique contribution of each independent variable within the model). The association between later bedtime and higher ISI scores during-lockdown was mediated by the later preferred time of day to train and daytime nap timing, lower training frequency and longer SOL. Also, the increased late night meals and lower training frequency mediated the relationship between longer SOL and higher ISI scores. The magnitude of the mediation effect is expressed as percentages according to the formula (indirect effect/total effect × 100).

Multiple linear regression models for PSQI [*F*_(5,1,675)_ = 89.78; *p* < 0.001; *R*^2^ = 0.22] and ISI [*F*_(5,1,675)_ = 38.21; *p* < 0.001; *R*^2^ = 0.11] were significant. The reduced sleep quality and increased insomnia severity from pre- to during-lockdown were negatively affected principally by the increase of SOL and the delayed bedtime (All *p* < 0.001). The results of the multiple regression and mediation models are presented in Figures 2, 3.

### The Effects of Ramadan Observance on Muslim Athletes’ Sleep Quality and Training Volume

**FIGURE 4:**
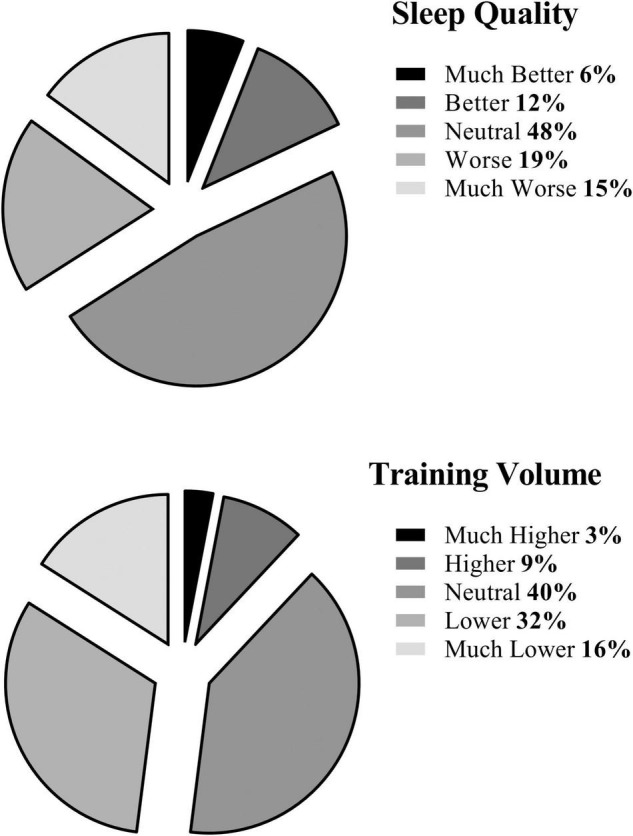
Muslim athletes were asked to subjectively rate their sleep quality (Much worse, Worse, Neutral, Better, and Much better) and their training volume (Much lower, Lower, Neutral, Higher, and Much higher) during-lockdown and Ramadan compared to lockdown outside of Ramadan.

The effect of Ramadan observance on sleep quality and training volume during-lockdown is presented in Figure 4. Both sleep quality (χ^2^ = 222.6; *p* < 0.001) and training volume (χ^2^ = 342.4; *p* < 0.001) were negatively affected by Ramadan observance. 32% of the Muslim athletes reported that both their sleep quality and training volume were lower; 38% reported that their sleep quality or training volume were lower during Ramadan; 25% reported that both their sleep quality and training volume were not affected by Ramadan observance; and only 5% reported better sleep quality and training volume when observing Ramadan during-lockdown compared to lockdown outside of Ramadan.

## Discussion

The original study (4) was the largest study investigating sleep and training behaviors within the context of COVID-19 related lockdown. The present data furthers this exploring Ramadan observance during lockdown, in an international sample of Muslim athletes. The main findings were that (***i***) Muslim athletes reported lower sleep quality and higher insomnia during- compared to pre-lockdown, (***ii***) Muslims were more affected by the lockdown compared to non-Muslim athletes, and (***iii***) Muslims athletes displayed lower sleep quality and training volume during-lockdown when observing Ramadan compared to during-lockdown outside of Ramadan.

Several reports showed disrupted circadian rhythms and reduced sleep quality during-lockdown in athletes (1–4), without taking into consideration the potential effect of Ramadan observance in addition to lockdown adherence. Indeed, Ramadan observance *per se* has been reported to reduce sleep quality and quantity in athletes and physically active individuals (11, 16) whilst lockdown has also been reported to reduce sleep quality and increase insomnia severity in athletes (1–4). We have shown that, the proportion of Muslim athletes reporting poor (68%) and very poor (32%) sleep quality during-lockdown was higher than that observed in the overall sample of athletes (49 and 22%, respectively) (4). Evidently, at times during the early 2020 lockdowns, the circadian rhythms of Muslim athletes were doubly challenged by lockdown and Ramadan observance whereas non-Muslim peers were only exposed to lockdown. Indeed, 70% of the current study’ sample reported that their sleep quality (34%) and/or their training volume (48%) were lower when observing Ramadan during-lockdown compared to during-lockdown outside of Ramadan. However, since the lockdown induced a delay in bedtime (+116 min) and wake-up time (+190 min) in the first place, a considerable proportion of Muslim athletes (30%) may have been normalized with the constraints superimposed by the lockdown, and were, therefore, not affected by Ramadan observance during-lockdown.

Muslim athletes reported longer (+7 min) and later daytime (+20 min) naps during- compared to pre-lockdown. These changes from pre- to during-lockdown were directly and indirectly associated with the changes in sleep quality and insomnia symptoms. Athletes could take naps for different reasons. Recuperative naps could be a replacement for lost sleep, prophylactic naps are used ahead of an expected sleep loss, and appetitive naps are just for the joy of napping (21). Short recuperative and appetitive naps could be beneficial for physical and cognitive performance (22–26). Long recuperative daytime naps (27), but not long appetitive naps (28), could also be beneficial for physical and cognitive performances. Although longer and later daytime naps have been associated with low sleep quality and high insomnia during-lockdown (4). The present and previous data (4) do not allow delineation of whether long daytime nap durations are a cause or a consequence of low nocturnal sleep quality. In a recent systematic review, Trabelsi et al. (16) reported an increase of the duration of daytime naps in individuals engaged in physical activity during Ramadan. Similarly, a 10-fold increase in the duration of daytime naps during Ramadan has been reported in soccer players (29) whilst increased frequency of daytime naps during Ramadan has been seen in junior-level Muslim athletes (30). Increased napping frequency and duration in fasting athletes may be related to the abstention from food consumption during the day, with napping employed to “kill-time,” until food and drink are permitted at sunset (i.e., *iftar*).

In Muslim majority countries, as per meals timing in general, the dusk meal (*iftar*) is a major *zeitgeber* during Ramadan, where all Muslim behaviors are scheduled relative to this. Further, Ramadan observance is characterized by an increased number of nocturnal meals, because food and drink consumption are only allowed between dusk and dawn; a major “first” main meal at dusk “*iftar*,” a secondary main meal near dawn “*suhur*,” and smaller meals/snacks in between. Indeed, the current sample of participants reported increased late-night eating behavior during- compared to pre-lockdown, which mediated the longer SOL and delayed bedtime, associated with the lower sleep quality and the higher insomnia during-lockdown. Actually, food consumption raises core body temperature and cortisol levels, in turn increasing alertness/arousal (31). Further, previous reports attributed the impairment of sleep quality during Ramadan to the increased consumption of fat and carbohydrate-rich foods (7, 32). Additionally, dietary changes, inducing gastrointestinal disorders during Ramadan, have been previously suggested to impair athletes’ sleep quality via sleep interruption (13). Also, caffeinated beverage consumption, increased during- compared to pre-lockdown. In this regard, caffeine consumption, even 6 h before bedtime, may still produce later bedtimes and longer SOL due to its long half-life (33).

Muslim athletes were training later during the day (+45 min) during- compared to pre-lockdown, which was associated directly and indirectly (i.e., through later bedtime) with the increase in insomnia severity. Moreover, lower training frequency during-lockdown mediated the effect of bedtime and SOL on insomnia severity. This change in training behavior (timing, frequency and duration) has been shown to impact sleep quality, especially the quantity of slow wave sleep (34). We indeed observed that the changes in training behavior during-lockdown were associated with lower sleep quality and higher insomnia in this sample of Muslim athletes. Besides, Muslim athletes observing Ramadan tend to split and/or spare their efforts through the day (6), and even during competitions (35). Indeed, 48% of the current sample of Muslim athletes reported lower training volume during Ramadan and lockdown compared to lockdown outside of Ramadan. Delaying and adapting training schedule/pattern might have been facilitated by the stop in major international competitions, putting less pressure on the athletes to maintain their usual busy training schedule. Besides, several reports showed that Muslim athletes naturally shift their training sessions near (i.e., 2–3 h before) or after (i.e., 2–3 h after) the *iftar* during Ramadan (6, 8, 35, 36), which could explain the later preferred TOD to train during-lockdown.

The current study showed that the early 2020 lockdown disrupted sleep (i.e., later bed and wake times and later and longer daytime naps), training (i.e., fewer and later training sessions) and eating (i.e., increased late night eating) behaviors in this international sample of Muslim athletes. Also, Ramadan observance reduced training loads and lowered sleep quality. Therefore, we showed that Ramadan occurring simultaneously to lockdown was a dual stressor to Muslim athletes, producing negative effects beyond lockdown alone. Although not investigated in the current study, the light saving hour (29 March to 26 October 2020 in the northern hemisphere) could have further disrupting effects on Muslim athletes’ sleep and training behaviors.

### Practical Applications

The current findings support the fact that Ramadan had an additional (and likely underestimated) disruptive and negative impact on Muslim athletes sleep and training behaviors. Therefore, more attention should be afforded to Muslim athletes when a circadian disrupter (e.g., lockdown, jet-lag) occurs during Ramadan. With the occurrence of a circadian disrupter, lockdown in this case, Muslim athletes and their coaching staff should enhance sleep hygiene strategies to hopefully protect the sleep-wake patterns (37), and *a priori* plan adequate training sessions (with appropriate frequency, volume and intensity) to optimize training/recovery (38).

### Limitations

The main limitations for the present study are that: (i) we had to collect data about the pre-lockdown period by retrospective self-report, which could be subjected to recall bias. Nevertheless, the study procedures are similar to other works evaluating sleep with surveys during the COVID-19 pandemic in athletes (1–3); (ii) the current study was advertised online and it could be subjected to sampling bias (i.e., non-Muslim, unintentionally or otherwise, answering the questions destined to Muslim athletes); (iii) the PSQI and ISI were used beyond their original purposes (thinking about the last month) and were adapted (pre- and during-lockdown comparison) to better suit the current study objective; and (iv) sleep and training were not evaluated objectively, but subjectively (self-reported data), using validated tools. Therefore, the current assumptions should be interpreted/extrapolated while taking these points into consideration.

## Conclusion

Muslim athletes’ reported lower sleep quality and higher insomnia severity during the early 2020 lockdown compared to non-Muslim athletes. In addition, sleep and training behaviors of Muslim athletes were further disrupted by Ramadan observance that occurred during-lockdown. With the greatest hope that the world population will not face additional spikes of COVID-19 cases (and associated lockdowns), these results provide important insights to better manage sleep and training in a population of international athletes.

## Data Availability Statement

All data are stored on institutional servers of the corresponding author and available on reasonable request.

## Ethics Statement

The studies involving human participants were reviewed and approved by the Farhat Hached Hospital, Sousse, Tunisia. The participants provided their written informed consent to participate in this study.

## Author Contributions

MR, KT, HC, and KC designed the study. MR performed the statistical analysis and prepared the first draft. AA, KT, JAV, MN, HBS, LT, SG, and KC critically revised the manuscript. All authors have been involved in the survey development, translation and data collection, and approved the manuscript’s final version.

## Conflict of Interest

The authors declare that the research was conducted in the absence of any commercial or financial relationships that could be construed as a potential conflict of interest.

## Publisher’s Note

All claims expressed in this article are solely those of the authors and do not necessarily represent those of their affiliated organizations, or those of the publisher, the editors and the reviewers. Any product that may be evaluated in this article, or claim that may be made by its manufacturer, is not guaranteed or endorsed by the publisher.

## Footnotes

1
gdpr-info.eu

## Orcid

Mohamed Romdhani, orcid.org/0000-0002-1715-1863; Achraf Ammar, orcid.org/0000-0003-0347-8053; Khaled Trabelsi, orcid.org/0000-0003-2623-9557; Hamdi Chtourou, orcid.org/0000-0002-5482-9151; Jacopo A. Vitale, orcid.org/0000-0002-7537-079X; Liwa Masmoudi, orcid.org/0000-0002-9628-1585; Mathieu Nédélec, orcid.org/0000-0001-8095-9163; Dale E. Rae, orcid.org/0000-0002-2584-6585; Ramzi A. Al Horani, orcid.org/0000-0002-6915-816X; Helmi Ben Saad, orcid.org/0000-0002-7477-2965; Nicola Bragazzi, orcid.org/0000-0001-8409-868X; Gürhan Dönmez, orcid.org/0000-0001-6379-669X; Ismail Dergaa, orcid.org/0000-0001-8091-1856; Tarak Driss, orcid.org/0000-0001-6109-7393; Abdulaziz Farooq, orcid.org/0000-0002-9162-4948; Omar Hammouda, orcid.org/0000-0002-5002-687X; Nesrine Harroum, orcid.org/0000-0003-2684-9785; Bahar Hassanmirzaei, orcid.org/0000-0003-2961-7955; Karim Khalladi, orcid.org/0000-0002-1522-4598; Syrine Khemila, orcid.org/0000-0001-5014-2865; Leonardo Jose Mataruna-Dos-Santos, orcid.org/0000-0001-9456-5974; Imen Moussa-Chamari, orcid.org/0000-0002-7849-9687; Iñigo Mujika, orcid.org/0000-0002-8143-9132; Hussein Muñoz Helú, orcid.org/0000-0001-9094-5566; Amin Norouzi Fashkhami, orcid.org/0000-0003-0139-4956; Laisa Liane Paineiras-Domingos, orcid.org/0000-0003-3451-5056; Mehrshad Rahbari Khaneghah, orcid.org/0000-0002-1392-3774; Yoshitomo Saita, orcid.org/0000-0003-1614-2575; Maher Souabni, orcid.org/0000-0002-9223-2305; Nizar Souissi, orcid.org/0000-0003-1227-8221; Jad Adrian Washif, orcid.org/0000-0001-8543-4489; Johanna Weber, orcid.org/0000-0002-3735-4254; Piotr Zmijewski, orcid.org/0000-0002-5570-9573; Lee Taylor, orcid.org/0000-0002-8483-7187; Sergio Garbarino, orcid.org/0000-0002-8508-552X; Karim Chamari, orcid.org/0000-0001-9178-7678.
