# Supplementary material for: Ramadan Observance Exacerbated the Negative Effects of COVID-19 Lockdown on Sleep and Training Behaviors: A International Survey on 1,681 Muslim Athletes
Source: Front Nutr. 2022 Jun 30;9:925092. doi: 10.3389/fnut.2022.925092 (PMC9283087; doi:10.3389/fnut.2022.925092)
Supplement: Supplementary file 1 [file Data_Sheet_1.docx]

**Supplementary File**

**Survey**

**Athletes' sleep pattern during the COVID-19 lockdown period**

**Preamble**

Globally, the coronavirus (COVID-19) pandemic is affecting/has affected peoples’ daily lives. Athletes are not immune to this, near all sporting events were suspended or canceled at some point, due to country/region-specific lockdowns. Other aspects of athletes' training, competition, and recovery programs have also been affected. This global survey will explore the impact of lockdown and/or restricted movement on athlete sleep patterns and some associated nutritional behaviors.

Your participation will enhance knowledge about athlete sleep patterns and some associated nutritional behaviors. The knowledge and data will be used for research purposes, specifically to inform current/future guidelines for athletes and their interdisciplinary supports networks (medical staff, coaches, sports scientists, parents, family, etc.)

All responses and data will be processed anonymously. You will not be asked to provide your name. Besides, no identifying information will be recorded, including IP address and email address. Only the principal investigator will have access to the dataset.

This international survey has been approved by the institutional review board of the Farhat HACHED University Hospital, Sousse, Tunisia (IRB N°: FH020720).

This questionnaire contains 47 questions and takes 10 ± 2 min to complete.

Please feel free to contact the principal investigator with any further questions about the survey or for details regarding the final results:

Mohamed Romdhani

Ph.D. in sports sciences, athletic sleep, and exercise physiology.

Tunisian National Sports Observatory

E-mail: [romdhaniroma@gmail.com](mailto:romdhaniroma@gmail.com)

By clicking on the following mark, **I certify that**;

- I am an athlete, competing at any given level
- I am at least 18 years old,
- I have been or I still in the lockdown,
- I have read and understood the survey’s instructions,
- I will answer the survey questions as accurately as possible,
- I declare my agreement to participate in this study.

**Click to confirm**

- **Tick**

**PART I: background and confinement information**

**Question 1:** Country of residence

……….

**Question 2:** City of residence

……….

**Question 3:** Gender

- Male
- Female
- Others
- Prefer not to say

**Question 4:** Age in years

……….

**Question 5:** Which sport you are competing in?

….....

**Question 6:** What is your level of practice in your sport?

- Amateur (not paid for practicing sport)
- Sub-elite (income from both sport and another activity)
- Elite (high level of sport practice, even when not paid for it)
- Professional (high level of practice with full income from sport)
- Others

**Question 7:** Number of training sessions per week

DURING THE LOCKDOWN

……….

DURING THE MONTH BEFORE THE LOCKDOWN

……….

**Question 8:** Are/were you keeping the same training intensity during compared to pre lockdown?

- Yes
- No
- I don’t know

**Question 9:** Are you still in lockdown?

- Yes
- No

**Question 10:** How long were you in lockdown?

| < 2 weeks | 2 – 4 weeks | 4 – 6 weeks | 6 – 8 weeks | > 8 weeks |
| --- | --- | --- | --- | --- |

**NB: Please use the 24h format (hh:mm; e.g., 17:37) when answering time questions**

**Question 11:** what is/was your preferred time of day (hh:mm; e.g., 17:37) to train

DURING THE LOCKDOWN?

…………….

DURING THE MONTH BEFORE THE LOCKDOWN?

**……….**

**PART II- Sleep Questionnaire; the adapted version of “the Pittsburg Sleep Quality Index”**

**Question 1a:** usual bedtime (hh:mm; e.g., 17:37)

DURING THE LOCKDOWN?

…………….

DURING THE MONTH BEFORE THE LOCKDOWN?

**……….**

**Question 2:** How long it takes you (in minutes) to fall asleep

DURING THE LOCKDOWN?

…………….

DURING THE MONTH BEFORE THE LOCKDOWN?

**……….**

**Question 3:** usual wake time (hh:mm; e.g., 17:37)

DURING THE LOCKDOWN?

…………….

DURING THE MONTH BEFORE THE LOCKDOWN?

**……….**

**Question 4:** hours of actual sleep per night (hh:mm; e.g., 17:37)

**NB: This may be different than the number of hours you spent in bed**

DURING THE LOCKDOWN?

…………….

DURING THE MONTH BEFORE THE LOCKDOWN?

**……….**

**Question 5.a:** DURING THE LOCKDOWN, how often have you had trouble sleeping because you;

|  | Not during the lockdown | Less than once a week | Once or twice a week | 3 or more time a week |
| --- | --- | --- | --- | --- |
| cannot get to sleep within 30 minutes |  |  |  |  |
| Wake up in the middle of the night or early morning |  |  |  |  |
| Have to get up to use the bathroom |  |  |  |  |
| Cannot breathe comfortably |  |  |  |  |
| Cough or snore loudly |  |  |  |  |
| Feel too cold |  |  |  |  |
| Feel too hot |  |  |  |  |
| Have bad dreams |  |  |  |  |
| Have pain |  |  |  |  |
| other reasons |  |  |  |  |

**Question 5.b:** DURING THE MONTH BEFORE THE LOCKDOWN, how often have you had trouble sleeping because you;

|  | Not during the month before the lockdown | Less than once a week | Once or twice a week | 3 or more time a week |
| --- | --- | --- | --- | --- |
| cannot get to sleep within 30 minutes |  |  |  |  |
| Wake up in the middle of the night or early morning |  |  |  |  |
| Have to get up to use the bathroom |  |  |  |  |
| Cannot breathe comfortably |  |  |  |  |
| Cough or snore loudly |  |  |  |  |
| Feel too cold |  |  |  |  |
| Feel too hot |  |  |  |  |
| Have bad dreams |  |  |  |  |
| Have pain |  |  |  |  |
| other reasons |  |  |  |  |

**Question 6.a:** DURING THE LOCKDOWN, how likely it was to use medication to help you sleep well?

| Not during the lockdown |
| --- |
| Less than once a week |
| Once or twice a week |
| 3 or more time a week |

**Question 6.b:** DURING THE MONTH BEFORE THE LOCKDOWN, how likely it was to use medication to help you sleep well?

| Not during the month before the lockdown |
| --- |
| Less than once a week |
| Once or twice a week |
| 3 or more time a week |

**Question 7.a:** DURING THE LOCKDOWN, how often had/have you trouble staying awake while driving, eating meals, or engaging in social activities?

| Not during the lockdown |
| --- |
| Less than once a week |
| Once or twice a week |
| 3 or more time a week |

**Question 7.b:** DURING THE MONTH BEFORE THE LOCKDOWN, how often had/have you trouble staying awake while driving, eating meals, or engaging in social activities?

| Not during the month before the lockdown |
| --- |
| Less than once a week |
| Once or twice a week |
| 3 or more time a week |

**Question 8:** how much of a problem has it been for you to keep up enough enthusiasm to get things done?

|  | DURING THE LOCKDOWN | DURING THE MONTH BEFORE THE LOCKDOWN |
| --- | --- | --- |
| No problem at all |  |  |
| Only a very slight problem |  |  |
| Somewhat of a problem |  |  |
| A very big problem |  |  |

**Question 9:** how would you rate your overall sleep quality?

|  | DURING THE LOCKDOWN | DURING THE MONTH BEFORE THE LOCKDOWN |
| --- | --- | --- |
| Very good |  |  |
| Fairly good |  |  |
| Fairly bad |  |  |
| Very bad |  |  |

**PART III: The adapted version of insomnia severity index**:

**Question 1.a:** Please rate the severity of your insomnia problem(s) DURING THE LOCKDOWN

|  | Difficulty falling asleep | Difficulty staying asleep | Problems waking up too early |
| --- | --- | --- | --- |
| None |  |  |  |
| Mild |  |  |  |
| Moderate |  |  |  |
| Severe |  |  |  |
| Very severe |  |  |  |

**Question 1.b:** Please rate the severity of your insomnia problem(s) DURING THE MONTH BEFORE THE LOCKDOWN

|  | Difficulty falling asleep | Difficulty staying asleep | Problems waking up too early |
| --- | --- | --- | --- |
| None |  |  |  |
| Mild |  |  |  |
| Moderate |  |  |  |
| Severe |  |  |  |
| Very severe |  |  |  |

**Question 2:** How SATISFIED are you with your sleep pattern?

|  | DURING THE LOCKDOWN | DURING THE MONTH BEFORE THE LOCKDOWN |
| --- | --- | --- |
| Very satisfied |  |  |
| Satisfied |  |  |
| Moderately satisfied |  |  |
| Dissatisfied |  |  |
| Very dissatisfied |  |  |

**Question 3.a:** DURING THE LOCKDOWN

|  | How NOTICEABLE to others do you think your sleep problem is in terms of impairing the quality of your life | How WORRIED are you about your sleep problem | To what extent do you consider your sleep problem to INTERFERE with your daily functioning (e.g. daytime fatigue, mood, ability to function at work/daily chores, concentration, memory, mood, etc.) |
| --- | --- | --- | --- |
| Not at all |  |  |  |
| A little |  |  |  |
| Somewhat |  |  |  |
| Much |  |  |  |
| Very much |  |  |  |

**Question 3.b:** DURING THE MONTH BEFORE THE LOCKDOWN

|  | How NOTICEABLE to others do you think your sleep problem is in terms of impairing the quality of your life | How WORRIED are you about your sleep problem | To what extent do you consider your sleep problem to INTERFERE with your daily functioning (e.g. daytime fatigue, mood, ability to function at work/daily chores, concentration, memory, mood, etc.) |
| --- | --- | --- | --- |
| Not at all |  |  |  |
| A little |  |  |  |
| Somewhat |  |  |  |
| Much |  |  |  |
| Very much |  |  |  |

**PART IV- Napping questions**

**Question 1:** How often do/did you nap?

DURING THE LOCKDOWN

…………..

DURING THE MONTH BEFORE THE LOCKDOWN

……………………

**Question 2:** If you did take nap, what is likely the duration of your naps?

DURING THE LOCKDOWN

…………..

DURING THE MONTH BEFORE THE LOCKDOWN

……………………

**Question 3:** What is/was your preferred time of day to nap?

DURING THE LOCKDOWN

…………..

DURING THE MONTH BEFORE THE LOCKDOWN

……………………

**PART V- Nutrition and health-related questions**

**Question 1:** Your weight

AT THE END OF THE LOCKDOWN

…………..

DURING THE MONTH BEFORE THE LOCKDOWN

……………………

**Question 2:** On average, how many meals do you eat daily?

DURING THE LOCKDOWN

…………..

DURING THE MONTH BEFORE THE LOCKDOWN

……………………

**Question 3:** Are you likely to eat after midnight?

|  | DURING THE LOCKDOWN | DURING THE MONTH BEFORE THE LOCKDOWN |
| --- | --- | --- |
| Very likely |  |  |
| Likely |  |  |
| Unlikely |  |  |
| Very unlikely |  |  |

**Question 4:** On average (weekdays and weekends), how many cups of coffee or caffeinated beverages do/did you drink daily?

DURING THE LOCKDOWN

…………..

DURING THE MONTH BEFORE THE LOCKDOWN

……………………

**Question 5:** On average (weekdays and weekends), how many cups of alcohol or alcoholic drink do/did you consume daily?

DURING THE LOCKDOWN

…………..

DURING THE MONTH BEFORE THE LOCKDOWN

……………………

**Question 6:** On average (weekdays and weekends), how many cigarettes do/did you smoke per day?

DURING THE LOCKDOWN

…………..

DURING THE MONTH BEFORE THE LOCKDOWN

……………………

**PART V- Ramadan questions**

**NB: To be answered only and solely by Muslim athletes fasting during 2020 Ramadan**

**Question 1:** How was your sleep quality when locked-down during Ramadan compared to the lockdown out of Ramadan?

| Much Worse | Worse | Same | Better | Much better | I don’t know |
| --- | --- | --- | --- | --- | --- |

**Question 2:** How was your training volume when locked-down during Ramadan compared to the lockdown out of Ramadan?

| Very lower | Lower | Same | Higher | Much Higher | I don’t know |
| --- | --- | --- | --- | --- | --- |
